# The 48-Hours Bill Applied to Working Nurses

**Published:** 1920-01-03

**Authors:** 

**Affiliations:** London Hospital, Whitechapel, E. 1.


### THE EDITOR'S LETTER-BOX.

**[Correspondence on all subjects is invited, but we cannot in any way be responsible for the opinions expressed by our correspondents, who must give their name and address as a guarantee of good faith, but not necessarily for publication. Correspondents are reminded that brevity of style and conciseness of statement greatly facilitate early insertion.]**

### THE 48-HOURS BILL APPLIED TO WORKING NURSES.

### Viscount Knutsford in Reply to Ierne.

*To the Editor of* The Hospital.

You write that you do not believe that any man or woman is physically able to work twelve hours a day, let alone twenty-four. But all private nurses at cases do so. A patient who has to undergo an operation engages only two nurses, and they divide the twenty-four hours between them into two shifts of twelve hours. In the ordinary sense of the word "work" it is not "work" for twelve hours, but none the less they are each responsible for the patient for twelve hours out of the twenty-four. If the forty-eight hours' Bill is to apply to nurses—and it will do so unless it be altered—a patient would have to engage three nurses. Q.E.D.—in other words, what I said.

Similarly, only worse, a mother engages only one nurse for her confinement, and that nurse is responsible for mother and baby for the whole twenty-four hours of every day and night. The mother, under the Bill, will have to engage three nurses.

I have never myself had a baby, but I have had two pretty severe operations, and I was killed, to all intents and purposes, in 1915. I never found any of my two nurses "exhausted," though I was never left alone during the twenty-four hours for several weeks. To talk of exhaustion is absurd. Two of the nurses I had had been doing this for twenty years.

Take district nursing. Is a nurse working in a district to stop after her eight hours, and another to go on with the round? It must be so if an employer is to be punished if he employs anyone for more than forty-eight hours. And what is to be done on Sundays? The forty-eight hours' Bill prescribes for a "six days' week" only. But patients do not recover on Sundays.

So I say that a forty-eight hours' week is impossible for nurses.

I said that we could not afford at the London Hospital to add 300 nurses to our staff and to build accommodation for them, which would be necessary if a forty-eight hours' Bill were passed for nurses. To this Ierne replies that, as I am a "blunt and plain man" (not my fault, but my misfortune), will I walk down the Thames Embankment and look at St. Thomas's, and I shall see what England can afford. I do not understand how this will help me. England did not build St. Thomas's; it was built from the proceeds of the sale of its City property given to its Governors in the back ages.—Yours faithfully,

Knutsford.

London Hospital, Whitechapel, E. 1.,
December 22, 1919.

